# Looking for COVID side effects in the EU through the analysis of health and behavioural profiles

**DOI:** 10.1007/s11135-022-01606-3

**Published:** 2022-12-30

**Authors:** Aurea Grané, Irene Albarrán, Diego Peran

**Affiliations:** Statistics Department, Universidad Carlos III de Madrid, C/ Madrid 126, 28903 Getafe, Spain

**Keywords:** Archetypoids, Care economy, COVID, Profiles, Wellbeing, 62H30, 62P25

## Abstract

More than two years after the great outbreak of COVID suffered in almost the whole world, and in particular in Europe, we have gradually learned about the direct effects of this virus on our health and what consequences it can have if we become infected. However, this pandemic also had great economic and social consequences that affected people in an indirect way, which we can call COVID side effects. In this work we carried out an innovative type of analysis based on the concept of archetypoids in order to find extreme observations in a database of mixed-type data and used them to classify individuals yielding to different health and behavioural profiles in coping with the COVID outbreak in the EU. We use data from the first COVID-19 Survey of the SHARE project (Survey on Health, Aging and Retirement in Europe). The resulting profiles are easier to interpret than others based on central observations, and help to understand how the situations of restrictions and lock-downs affected people since the outbreak of the pandemic. Another key point of the work was to analyse how determinant are some aspects such as gender, age group or even geographical location in how each person experienced the pandemic. The method that we propose is wide enough to be used in other health and wellbeing surveys.

## Introduction

In December 2019, the first outbreak of pneumonia of unknown causes was detected in Wuhan, China. The rapid spread of the disease caused the World Health Organization (WHO), on January 30, 2020, to declare it a health emergency of international concern. As of that date, the disease had been detected in all provinces of mainland China, 57 and cases were diagnosed in 15 other countries (Bueno-Notivol et al. 2021). On March 11, 2020, a pandemic situation was declared internationally by WHO due to COVID-19 since the disease was already found in more than 100 territories worldwide (WHO 2020).

The COVID-19 is an infectious disease caused by SARS-CoV-2. The official names COVID-19 and SARS-CoV-2 were issued by WHO on 11 February 2020: CO for corona, VI for virus, D for disease and 19 because the outbreak was first identified in 2019. As it is a new virus, several of its characteristics are completely unknown, such as the symptoms it causes, its spread or its treatment. Today we already know, that symptoms of COVID-19 often include fever, cough, headache, fatigue, breathing difficulties, and loss of smell and taste. The declaration of a pandemic and the measures taken by different countries due to the critical health situation in which they were, caused a complete change in lifestyle, habits and social relationships in people’s lives. Measures of social distancing, closure of public establishments, use of masks or even lock-downs were established in several countries of the world (Scherpenzeel et al. 2020).

Despite the fact that the appearance of this virus caused a global pandemic that affected practically all parts of the world, our work and data are focused on people aged 50 or older, living in the European Union (except Austria, plus Israel), which in total add up to 27 countries. This represents a total of approximately 187.2 million people, since the European Union, after the departure of Great Britain, has approximately 185 (without taking into account the population of Austria) million inhabitants and Israel with 2.2 million inhabitants (Eurostat 2020). This amount of people represents approximately $6\%$ of the world population and, above all, a large part of the most developed countries in the world are represented, so this study will help us to know how the pandemic affected the older citizens of these countries.

After the March 2020 outbreak and the time of strong restrictions and lock-downs experienced in many European countries, it is common to ask questions about how this virus and the restrictions affected on the European population, how these consequences changed depending on the age group, gender or country. Therefore, it is interesting to know what profiles arose since the appearance of the virus, what characteristics they have, regarding health and social behaviour, and how they are geographically distributed. For this purpose we analysed the first SHARE COVID-19 Survey (Börsch-Supan 2020), which was undertaken on people aged 50 and over, and consists of different questions related to respondents’ physical and mental health, changes in their social life and in their healthcare, and how closely COVID-19 affected them or their families. In particular, we propose a protocol to find and analyse profiles based on extremal observations, through archetypoidal analysis (Vinué et al. 2015). The advantage of this non-supervised statistical learning technique in front of other well-known clustering procedures like *k*-means or PAM (partitioning around medoids), is that profiles based on extremal individuals are easier to interpret than those based on central individuals, in the sense that it is easier to get a picture of a group consisting of “individuals with the worst health” rather than “individuals with intermediate health”.

The protocol is based on three stages: First, composite indicators are designed, each of which focuses on a particular topic of interest, such as general health, physical health, mental health, fear to leave home, practice of safety measures, social isolation, electronic isolation, experience with COVID-19, and changes in healthcare; Second, archetypoidal analysis is applied on these indicators to obtain a few set of representative individuals, and next profiles are created according to them; Finally, the resulting clusters are analysed and visualized by means of other socio-demographic variables of interest, not included in the design of the indicators. The method can be applied to large datasets with multivariate heterogeneous data, and it is wide enough to be used in other wellbeing or health surveys.

The paper proceeds as follows. In Sect. 2 we provide some related work on COVID side effects on mental health, as well, as similar analyses on SHARE or other datastes. In Sect. 3 we give a brief description of archetypoidal analysis, which is used to search for a set of representative individuals, next we detail the profile definition and the final clustering. In Sect. 4 we describe the database for the application, and more details are given on the design of composite indicators within this database. Section 5 contains the analysis of the results, and main conclusions are given Sect. 6.

## Literature review

In this Section we provide a non exhaustive literature review on the effects of COVID-19 on mental health, as well as some recent works that use SHARE COVID-19 database to show evidence of pandemic side effects on mental health.

COVID-19 pandemic has caused dramatic effects on population’s health and well-being. Social distancing strategies, which were crucial for limiting the spread of the virus and alleviating pressure on health systems, altered drastically people’s lifestyles producing socio-psychological discouraging consequences. Some studies stated that social isolation increased the risk of mental health problems such as depression (Santini et al. 2020; Litwin and Lewinski 2021); Pfefferbaum and North (2020) found that those who have or had COVID-19-related symptoms are more likely to develop general psychiatric disorders and are lonelier and Zixin and Wang (2020) pointed out that women and young people have higher risks of general psychiatric disorders and loneliness, while having a job and living with a partner are protective factors.

A larger negative psychological impact has been linked to factors including extended periods of quarantine, fears of infection, lack of knowledge on the virus, financial difficulty, and job loss (Bueno-Notivol et al. 2021). Anxiety and depression can become a barrier to rational medical and mental health interventions during pandemics as people are exposed to uncontrollable events, they exhibit helplessness and lack of motivation (de Bruin 2021). Among the extended literature, other related works that reach to similar conclusions are Rodríguez-Hidalgo et al. (2020), Codagnone et al. (2020), Moreno et al. (2020), Mata et al. (2021), Martinez-Garcia et al. (2021) and Martinez-Garcia et al. (2022).

Regarding SHARE COVID-19 database, by the time this paper was written, more than a hundred research papers were produced. Some of the most recent works related to mental health are Atzendorf and Gruber (2021), Bogdanova and Vladimirov (2021) and García-Prado et al. (2022). A complete list can be found at http://www.share-project.org/share-publications/share-covid-19-publications.html.

## Methodology

Multivariate heterogeneous data comprise both numeric and categorical features, and mixed datasets frequently occur in socio-demographic surveys as well as in other disciplines, such as economics, health, finance, marketing, etc. One of the challenging aspects of dealing with multivariate heterogeneous data is to find structures and to group similar individuals for further analysis. Additionally, the availability of large and very large datasets adds complexity to this matter.

In this work we design a protocol to search for and analyse clusters based on extremal observations, that can be applied to large datasets with multivariate heterogeneous data, with the advantage that the resulting profiles are easier to interpret than those based on central individuals, in the sense that it seems easier to understand a group formed by individuals with “the worst” characteristic of interest rather than a group whose individuals take “intermediate values” in that characteristic.

In what follows we detail the protocol that we propose, which is based on three stages. More details are given in Sect. 4, where the methodology is illustrated through the SHARE COVID-19 database (Börsch-Supan 2020) with the aim of searching for different health and behavioural profiles among older Europeans that arose due to the COVID-19 pandemic.

### First stage

In this stage, the survey information is split into two groups: descriptive variables and thematic blocks. Next, indicators are designed within each thematic block.

The group of descriptive variables will not contribute to the indicators and it is left unchanged. The kind of variables to be included in this group is usually related to socio-demographic information such as country, gender, age group, etc.

Variables within thematic blocks may focus on a single aspect or characteristic of interest. For each thematic block, a composite indicator is constructed by addition of the variables within each block, and rescaled to 0–10. It is recommended that variables within thematic blocks are encoded in a similar way and maintain the same polarity. In Sect. 4 we design nine thematic indicators according to the information contained in the SHARE COVID-19 database.

### Second stage

Once thematic indicators are constructed, in the next stage we apply archetypoidal analysis on them with the aim of searching for a few, say *k*, observed, extremal cases o pure types, called archetypoids. Once these $k$ individuals are found, the next step is to partition the dataset around them giving rise to $k$ groups. In particular, each observation in the dataset is associated to the closest archetypoid, in this way the archetypoid is the representative observation of each group or profile. That is, the characteristics of each archetypoid are those of the group it represents.

#### Archetypoidal analysis

Archetypoidal analysis is a particular case of archetypal analysis, where each individual in a database is represented as a mixture of individuals considered as extreme cases or archetypes. The archetypes themselves are restricted to be mixtures of the individuals in the dataset while the archetypoids are actual observations of the sample. That is, instead of being a mixture of several observations, an archetypoid is only one observation (it is itself). The computation of archetypes or archetypoids is a complex non-linear least squares problem that is solved using a optimization algorithm.

The concept of archetypes was introduced in Cutler and Breiman (1994). From that moment on, this type of analysis was applied in different fields such as market research, biology, genetics, sports, industrial engineering and different machine learning problems. Later, it was found that in some studies it was necessary for the computed archetype to be a real observation of the dataset, that is, that some data from the sample fits this archetype completely. That is why the concept of archetypoid was introduced (Vinué et al. 2015).

In what follows we give a brief summary of the fundamentals of archetypoidal analysis. See Vinué et al. (2015) for more details.

Let ${\mathbf {X}}$ be a matrix of dimensions $n\times m$, that contains the measurements of the *m* quantitative variables (in our case, the composite indicators) on *n* individuals, and we denote its rows by ${\mathbf {x}}_i$. The objective of archetypoidal analysis is to find a matrix ${\mathbf {Z}}$ of dimensions $k\times n$, whose rows (denoted by ${\mathbf {z}}_j$) are the *k* archetypes in the dataset, in such a way that data can be approximated by mixtures of the archetypes. To obtain the archetypes, we need to compute two matrices ${\mathbf {A}}=(\alpha _{ij})_{\{1\le i\le n, 1\le j\le k\}}$ and ${\mathbf {B}}=(\beta _{jl})_{\{1\le j\le k, 1\le l\le n\}}$ which minimize the residual sum of squares (RSS) that arises from combining the equation where ${\mathbf {x}}_i$ is approximated by a linear combination of ${\mathbf {z}}_j$’s (archetypes), $\sum _{i=1}^{n}\Vert {\mathbf {x}}_i-\sum _{j=1}^k\alpha _{ij}{\mathbf {z}}_j\Vert ^2$, and the equation where ${\mathbf {z}}_j$’s is expressed as a linear combination of the data, ${\mathbf {z}}_j=\sum _{l=1}^n\beta _{jl}{\mathbf {x}}_l$:

$$\begin{aligned} RSS=\sum _{i=1}^n\Vert {\mathbf {x}}_i-\sum _{j=1}^{k}\alpha _{ij} {\mathbf {z}}_j\Vert ^2=\sum _{i=1}^n\Vert {\mathbf {x}}_i -\sum _{j=1}^k\alpha _{ij}\sum _{l=1}^n\beta _{jl}{\mathbf {x}}_l\Vert ^2, \end{aligned}$$

under the constrains

(i) $\sum _{j=1}^k \alpha _{ij}=1$, with $\alpha _{ij} \ge 0$ for $i=1,\ldots ,n$, and
(ii) $\sum _l \beta _{jl}=1$, with $\beta _{jl}\in \{0,1\}$ and $j=1,\ldots ,k$.

To summarize, archetypoids should satisfy two conditions: all observations in the dataset can be approximated by convex combinations of the estimated archetypoids, and all archetypoids must be some observation in the dataset. The second condition differentiates archetypoids from archetypes, which need not be observed individuals of the dataset. In our case, the extreme cases that we search for represent specific persons, whose characteristics will describe those of their associated group.

As any unsupervised learning technique, before starting the archetypal analysis, it is necessary to choose the value of $k$, that is, the number of archetypoids to search for. Depending on the dimensions and characteristics of the dataset, the optimal value will be different. The objective is to find a value for *k* that minimizes the RSS as much as possible, but at the same time this optimal value will also depend on the type of analysis we want to do after finding these extreme cases. To assist in this decision, a scree-plot can be produced by representing the RSS in front of *k*, and use the “elbow” method to take the final decision. In our case, since the purpose is to create different profiles in the European Union and our dataset does not contain too many variables (only nine composite indicators), the optimal value of archetypoids to be calculated should not be high.

Once the optimal number of archetypoids is chosen, the computation of the archetypoids proceeds by implementing the algorithm by Vinué et al. (2015), which is based on PAM clustering algorithm, and consists of two steps: BUILD and SWAP. In the first step, an initial set of arquetypoids is computed and, in the second one, an attempt is made to improve this initial set by changing the observations chosen by others not selected, and checking if these replacements reduce RSS. The algorithm is implemented in the R-packages anthropometry (Vinué 2017) and adamethods (Vinué and Epifanio 2021), the latter being very useful when working with large datasets, since subsampling is used to reduce the computational cost.

#### Allocations

Once the *k* archetypoids are obtained, the next step is to partition the dataset around these extreme observations to create *k* groups or profiles.

One possibility is to use archetypoidal analysis results to make the allocations by assigning each individual to the profile with maximum $\alpha$ value. Even for moderately large datasets ($\ge 10^4$), where the dimensionality problem is circumvented with subsampling, to allocate individuals using the $\alpha$’s is still feasible by using function do_alphas_rss from adamethods R package.

Another possibility is to use a geometrical criterion an to assign each observation of the dataset to the closest archetypoid by means of a proper distance. In this case, since all variables (composite indicators) are of quantitative type and pairwise correlated, we will use Mahalanobis distance. In particular, given the set of *k* archetypoids $\{{\mathbf {z}}_1,\ldots ,{\mathbf {z}}_k\}$, observation ${\mathbf {x}}_i$ is assigned to the group defined by archetypoid ${\mathbf {z}}_j$ if

$$\begin{aligned} {\mathbf {z}}_j=\mathop {{\mathrm{arg\ min}}}\limits _{1\le l\le k} d_M^2({\mathbf {x}}_i,{\mathbf {z}}_l) =\mathop {{\mathrm{arg\ min}}}\limits _{1\le l\le k}({\mathbf {x}}_i-{\mathbf {z}}_l)'{\mathbf {S}}^{-1}({\mathbf {x}}_i-{\mathbf {z}}_l), \end{aligned}$$

where ${\mathbf {S}}$ the covariance matrix of ${\mathbf {X}}$.

### Third stage

Once the $k$ groups are obtained, we propose to analyse their distributions in terms of different descriptive variables, such as gender, age group and country. Remind that each computed archetypoid represents its group or profile, that is, the characteristics of that archetypoid (the values it takes for each composite indicator) define the characteristics of the associated group. It may be also of interest to know the frequencies of each profile, that is, if there are groups much more frequent than others within the population. Moreover, a brief individual analysis of each profile or group should be performed to know their properties. That is, to know what values the individuals that compose them take in the different indicators and to check if these characteristics of the group are similar to those of the archetypoid that yield them. It may also be of interest to visualise the geographical distribution of the profiles.

For instance, in Sect. 5 we will see that the profiles obtained with this methodology allow us to know how the first wave of COVID affected the population based on gender, age group or geographical location. That is, countries are classified by how the outbreak affected their citizens and what consequences they suffered or how it affected each age range of the European population. Finally, within the *k* archetypoids there are some that correspond to individuals with “bad” values in some indicators, such as poor health, poor mental health or high social isolation, so it is interesting to see how these groups with high values are distributed by the population and by country.

## Data and methods

In this Section we start by describing the dataset where the methodology is applied, and the construction of nine composite indicators follows.

### The SHARE COVID-19 database

The Survey of Health, Ageing and Retirement in Europe (SHARE) is a multidisciplinary and cross-national collection of databases of micro data in different fields like health, socio-economic status and social and family networks. These surveys contain samples of individuals aged 50 or over, and cover all the countries of the European Union including Switzerland and Israel (Börsch-Supan 2020). In this work, we use the database called SHARE COVID-19 survey, that contains data collected via computer-assisted telephone interviews between June and August 2020^1^, and consists of 52,310 observations corresponding to each of the surveyed individuals and 241 columns or variables. Moreover, each observation in the database has a calibrated weight, so that weighted survey estimates match the known population totals. These weights have been taken into account in all the computations and graphs of our study (Prasad et al. 2017).

**Fig. 1:**
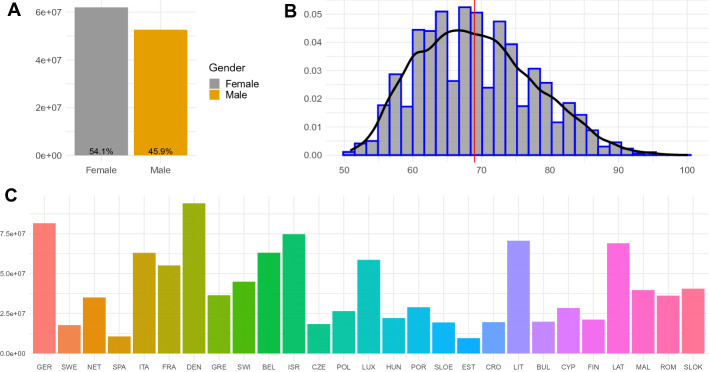
Distribution of gender, age and country in the database

In Fig. 1 we show the distribution of gender, age and country of the surveyed individuals, where we can see that 54.1% are female, and the median age is 68.8 years old.

The variables considered in the survey try to collect information of the individual of all kinds, and the most important life domains of the target population are *Health*: General health and changes in health since the COVID-19 outbreak including, in particular, variables that measure the physical health of the respondent; *Health behaviour*: Practice of safety measures or different habits that became common since the appearance of the virus such as social distancing or wearing a mask; *Mental health*: Variables that measure anxiety, depression, sleeping problems, and loneliness and how it changed since the COVID outbreak; *Infections and healthcare*: How closely COVID directly affects the respondent and how it changed the respondent’s healthcare; *Changes in work and economic situation*: Unemployment, business closures, working from home, changes in working hours and income or financial support; and *Social networks*: If the individual’s social relationships or contact with family or friends changed and how they changed (use of electronic devices).

### Design of thematic indicators

In order to obtain the profiles efficiently, nine indicators were designed from 35 variables listed in Table 1. The data was grouped by variables that dealt with (1) the respondents’ general health, (2) their physical health, (3) their mental health, (4) their fear of leaving home, (5) their practice of safety measures, (6) their social isolation, (7) their electronic isolation, (8) their experience with COVID, (9) their healthcare.

During the data processing task it was noted an excessively high percentage of missing values in variables related to the economy. This is the reason why we decided not to take into account this group of variables for the subsequent analysis. Thus, we decided to create the profiles using the information of the most relevant variables, regarding percentage of response and variability of responses.

**Table 1:** Variables included in the analysis

Description	Values/Categories
How was your health before outbreak	1-Excellent/2-Very good/3-Good/4-Fair/5-Poor
Change in your health since outbreak	0-Improved/1-About the same/2-Worsened
Falling down more in last 6 months	1-Yes/0-No
Fear of falling down in last 6 months	1-Yes/0-No
Dizziness, faints or blackouts in last 6 months	1-Yes/0-No
Fatigue in last 6 months	1-Yes/0-No
Felt nervous in last month	1-Yes/0-No
Sad or depressed in last month	1-Yes/0-No
Trouble sleeping recently	1-Yes/0-No
How often do you feel lonely	0-Hardly ever or never/1-Some of the time/2-Often
Went shopping since outbreak	−1-More often/0-About the same/1-Less often/2-Not any more
Went out for a walk since outbreak	−1-More often/0-About the same/1-Less often/2-Not any more
Met more than 5 people outside household since outbreak	−1-More often/0-About the same/1-Less often/2-Not any more
Visited other family members since outbreak	−1-More often/0-About the same/1-Less often/2-Not any more
Wore a face mask in public	0-Never/1-Sometimes/2-Often/3-Always
Kept distance from others in public	0-Never/1-Sometimes/2-Often/3-Always
Washed hands more than usual	1-Yes/0-No
Used hand sanitizer or desinfection fluids more than usual	1-Yes/0-No
Covered coughs and sneezes more than usual	1-Yes/0-No
Took drugs or medicine as prevention against COVID	1-Yes/0-No
Contact frequency with own children	5-Never/4-Less often/3-About once a week/2-Several times a week/1-Daily
Contact frequency with other relatives	5-Never/4-Less often/3-About once a week/2-Several times a week/1-Daily
Contact frequency with neighbours, friends or colleges	5-Never/4-Less often/3-About once a week/2-Several times a week/1-Daily
Electronic contact frequency with own children	5-Never/4-Less often/3-About once a week/2-Several times a week/1-Daily
Electronic contact frequency with other relatives	5-Never/4-Less often/3-About once a week/2-Several times a week/1-Daily
Electronic contact frequency with neighbours, friends or colleges	5-Never/4-Less often/3-About once a week/2-Several times a week/1-Daily
Anyone had COVID symptoms	1-Yes/0-No
Anyone tested positive for COVID	1-Yes/0-No
Anyone hospitalized due COVID	1-Yes/0-No
Anyone died due to COVID	1-Yes/0-No
Forwent medical treatment since outbreak	1-Yes/0-No
Postponed medical appointment due to COVID	1-Yes/0-No
Denied medical appointment since outbreak	1-Yes/0-No
Treated in hospital since outbreak	0-Yes/1-No
Visited medical facilities other than hospital	0-Yes/1-No

Thematic indicators were designed and created as follows: First, variables listed in Table 1 were grouped according to a topic of interest; Second, indicators were obtained by addition within topics, and finally rescaled to 0-10. Before variable addition, some changes in the original variable values were done, so that all variables contributing to the indicators followed the same polarity. In particular, the higher the values of the indicator the worse the individual’s situation since the outbreak.

We start by describing which variables were used in the creation of which indicator, and general description of the indicators follows. It is also of importance how each indicator affects each group or sector of society. Thus, in the Appendix we provide some plots of their distributions by gender, age (split into three groups: 50–65, 66–80, 80+), and country.

- *General health* How was your health before outbreak; Change in your health since outbreak.
- *Physical health* Falling down more in last 6 months; Fear of falling down in last 6 months; Dizziness, faints or blackouts in last 6 months; Fatigue in last 6 months.
- *Mental health* Felt nervous in last month; Sad or depressed in last month; Trouble sleeping recently; How often do you feel lonely.
- *Stay at home* Went shopping since outbreak; Went out for a walk since outbreak; Met more than 5 people outside household since outbreak; Visited other family members since outbreak.
- *Practice of safety measures*: Wore a face mask in public; Kept distance from others in public; Washed hands more than usual; Used hand sanitizer or disinfection fluids more than usual; Covered coughs and sneezes more than usual; Took drugs or medicine as prevention against COVID.
- *Social contact* Contact frequency with own children; Contact frequency with other relatives; Contact frequency with neighbours, friends or colleges.
- *Electronic social contact* Electronic contact frequency with own children; Electronic contact frequency with other relatives; Electronic contact frequency with neighbours, friends or colleges.
- *COVID* Anyone had COVID symptoms; Anyone tested positive for COVID; Anyone hospitalized due COVID; Anyone died due to COVID.
- *Healthcare* Forwent medical treatment since outbreak; Postponed medical appointment due to COVID; Denied medical appointment since outbreak; Treated in hospital since outbreak; Visited medical facilities other than hospital.

####  General health indicator

It informs about the person’s general health. A high value indicates poor health of the individual and vice versa. First of all, remind that surveyed people are all aged 50 or over, and therefore their general health is not the best in general. For this reason, this indicator very usually takes values greater than 3 or 5. As we can see in Fig. 9 in the Appendix, it presents a small variability depending on the age group where the two oldest age groups take the highest values, and the worst health. On the other hand, there does not seem to be any apparent distinction in terms of gender. Regarding countries, France and Romania stand out a bit with low values, but in general there are no great differences among countries.

####  Physical health indicator

It informs about the physical health of the individual. A high value indicates poor physical health of the individual and vice versa. Figure 10 in the Appendix contains its distribution by gender, age group and country. As was the case with General health indicator, when dealing with elderly people, this indicator takes higher values than normal, although due to the variables that make up it, the youngest people on slopes should not have high values. The greatest values are attained by people aged 80 or older, as might be expected. There are also no differences in terms of gender or in terms of countries, some stand out, such as Spain, Luxembourg, Estonia, with better physical health than the others and, on the contrary, Bulgaria with a remarkably high value.

#### Mental health indicator

It informs about psychological or mental health. A high value indicates poor mental or psychological health of the individual and vice versa. Figure 11 in the Appendix contains its distribution by gender, age group and country. Unlike the other two health indicators, this one in particular is not so related to age, since being very old does not affect having good psychological health as much as it does affect physical health. There do not seem to be differences based on age, but there are differences on gender, where women have worse mental health than men. On the other hand, several of the countries that had good physical health also have good mental health, such as Spain or Latvia. However, Bulgaria stands out above the rest for its worse mental health.

#### Stay at home indicator

Its tries to measure the fear of the respondent of going out from home. A high value indicates that the individual has hardly left home since the outbreak and vice versa. Figure 12 in the Appendix depicts its distribution by gender, age and country. In general, it shows greater variability than those related to health, perhaps due to the different restrictions lived in each country. Later in the paper we will present the stringency index that will help us to better understand the situation that each country experienced in the first wave of the pandemic, and thus understand the values of this indicator in some countries. It is clear that older people, of the second and third age groups, have stayed longer at home since the outbreak and women more than men. In terms of the country, the Nordic countries like Sweden or Denmark tend to take low values.

#### Practice of safety measures indicator

It informs about the change in habits since the appearance of COVID. A high value indicates that the individual changed their habits and maintain safety measures since the outbreak and vice versa. Its distribution by gender, age and country is shown in Fig. 13 in the Appendix, where no great differences are observed depending on age or gender. Nevertheless there is great variability in terms of countries, where countries such as Italy or Sweden stand out where many health measures were practised. This finding may indicate that there are no great differences between individuals within countries but the differences are noticeable between countries.

#### Social contact indicator

It tries to measure the social isolation of the respondent. A high value indicates that the individual did not have contact with family, friends or acquaintances and vice versa. Fig. 14 in the Appendix contains its distribution by gender, age group and country. As might be expected in this case, there seems to be a change in age groups as older people had less social contact as they were at risk. Regarding countries, Portugal and Estonia stand out as those that had the least social contact, and therefore their citizens were more socially isolated. It is interesting that this indicator does not take values similar to those of the Practice of safety measures indicator, where intuitively people of the countries that experienced a strict lock-down should be more careful with the virus. This finding may indicate that although some countries imposed restrictions, their citizens did not take as many precautionary measures against COVID and vice versa.

#### Electronic social contact indicator

With this indicator we want to measure the isolation of the respondent regarding electronic devices. Although both this and the previous indicator measure the individual’s social contact, it is interesting to see if there are differences depending on the type of contact (physical or electronic) that they had with their family or friends. Moreover, this indicator can be of special importance on countries that established strict lock-downs, where can help us to analyse to what extend electronic devices were used by the most vulnerable groups. A high value indicates that the individual has not maintained contact with family friends or acquaintances through electronic devices and vice versa. In Fig. 15 in the Appendix we show its distribution by gender, age group and country, where we observe that its behaviour in the population is similar to that of Social contact indicator. As expected, an important change is observed in the age ranges since the youngest maintained more social contact through electronic devices, probably because they were more used to using them.

####  COVID indicator

It informs about whether the individual or their relatives had direct contact with COVID. A high value indicates that COVID directly affected the respondent or someone close to him/her and vice versa. Its distribution by gender, age group and country is provided in Fig. 16 in the Appendix. It was expected that this indicator would take the value 0 in most cases, since the majority of the European population did not have direct contact with the virus at the time this survey was conducted (by the end of the first pandemic wave), although in some countries there were many more infections than in others. The only striking thing are the seven countries that have higher values than the others, which may indicate that the citizens of these countries suffered a lot during the first wave of the pandemic.

####  Healthcare indicator

It measures the level of difficulties in healthcare received by the individual since the outbreak. A high value indicates that the individual’s healthcare was deteriorated and vice versa. Figure 17 in the Appendix shows its distribution by gender, age group and country. A possible explanation of the surprising differences according to age may be that people in the two oldest groups generally have more medical appointments than the youngest, and therefore, with the outbreak, they were denied or postponed. On the contrary, there do not seem to be differences by gender and in terms of the countries there is not a great variance in general. In any case, this indicator is very useful to know the situation of each country during the first wave and how its health system was affected.

## Results

In this Section we analyse the results obtained when applied the methodology described in Sect. 3 to the SHARE COVID-19 database.

To choose an appropriate number *k* of archetypoids we applied archetype analysis by using the anthropometry R-package, and calculated the residual sum of squares (RSS) for $k=1,\ldots ,14$. The scree-plot with the resulting values is provided in Fig. 18 in the Appendix. With the aim of searching for the lowest value of RSS and taking into account the small number of variables (only nine composite indicators), we decided to take $k=6$.

### Searching for the arquetypoids

To compute the archetypoids, we used the $\texttt {adalara no paral}$ function from the adamethods R-package to reduce the computational cost of the technique when applied to large datasets. This function estimates the archetypoids via subsampling from a given number *N* of random subsamples of size $m=10$. We computed *N* using the following formula:

$$\begin{aligned} N=\left\lfloor 1+\frac{n-m}{m-k}\right\rfloor , \end{aligned}$$

where $\lfloor \cdot \rfloor$ denotes the integer part, *n* is the size of our database, that is, the rows of matrix ${\mathbf {X}}$, and *k* is the number of archetypoids we want to look for. When applying this formula to $m=10$, $n=33219$, $k=6$, we got $N=8303$. The selected archetypoids are shown in Table 2.

**Table 2:** Arquetypoids

n	Gener. health	Phys. health	Mental health	Stay home	Safety measures	Social contact	E-Soc. contact	COV	Health-care
Arch.1	8.3	7.5	10	9.2	9	8.7	7.3	0	4
Arch.2	5.0	0.0	0	10.0	9	10.0	5.3	0	4
Arch.3	8.3	7.5	0	7.5	4	6.7	4.7	0	4
Arch.4	1.7	0.0	0	3.3	4	6.7	8.7	0	4
Arch.5	5.0	0.0	0	3.3	9	4.7	2.7	0	4
Arch.6	5.0	0.0	10	9.2	8	7.3	3.3	10	6

Within this group of 6 archetypoids there are individuals with different characteristics each, with different values in each indicator. In short, Arch.1 and Arch.3 represent individuals with poor health compared to Arch.4 who is in good health; On the other hand, Arch.6 maintained very little social contact while Arch.4-5 did not suffered as much in this appearance. It is worth highlighting Arch.6, since it is the only one that scores more than zero in the COVID indicator and therefore suffered from this disease closely, and also the healthcare indicator scores higher than the others. On the other hand, Arch.4 stands out for having low values in almost all indicators and therefore for not having physical or mental health problems or lack of social contact.

In conclusion, each archetypoid has its own characteristics and these will be the ones that define the group or profile that each one generates. However, it is evident that in our study archetypoids such as Arch.6, that represents an individual in bad conditions, will have more relevance, that is, we will see how the most extreme observations are distributed, both good and bad.

### From archetypoids to profiles

**Fig. 2:**
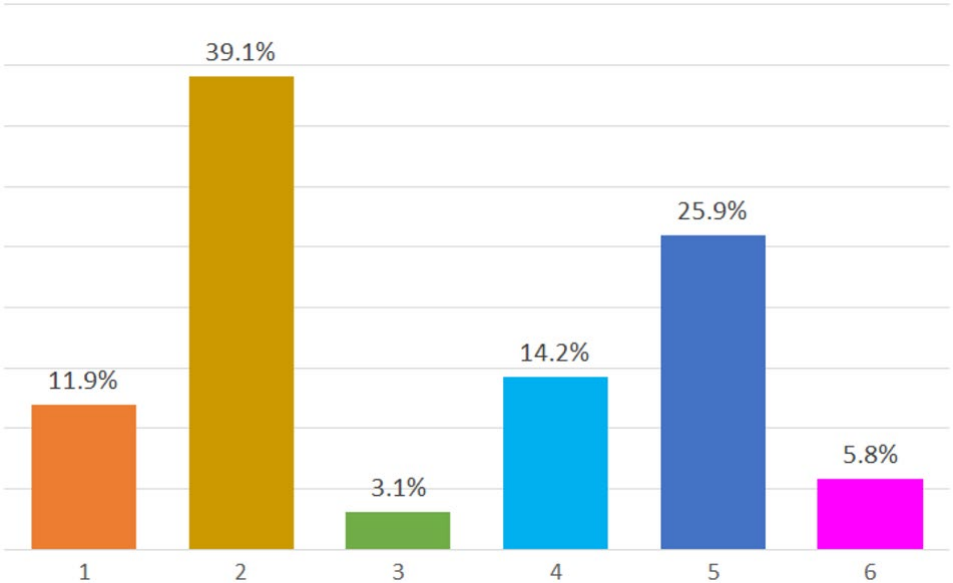
Frequency distribution of the profiles

As we commented before, it is time to create the different profiles from the selected archetypoids. For this purpose we use the Mahalanobis distance and assign each observation in the database to its closest archetypoid. In Fig. 2 we show the distribution of these 6 groups or profiles by means of a frequency graph. Taking into account that the total population of the 27 countries surveyed corresponding to EU Member States (except Austria) and Israel represent 448 million inhabitants (Eurostat 2020), (OECD 2012), we can estimate how many people there are of each profile in these countries.

In first place, there is profile 2, which represents a total of 73.2 million inhabitants, followed by profile 5, which represents 49 million people. Profile 2 corresponds to the individuals closest to Arch.2 that represented a person whose social life had been greatly reduced since the outbreak, so it makes sense that this is the most common profile in the European population. However, profile 5, whose archetypoid represents an individual who, despite the pandemic, had continued to maintain social contact and left home regularly, is the second most frequent. In second place, there are profiles 4 and 1 that represent, respectively, 26 and 22 million inhabitants each. They correspond to two opposite archetypoids: the former with good health, little fear of leaving home and little change in habits since outbreak and the latter quite the opposite. However, these two profiles present similar frequencies in the population, being completely different. Finally, the two least frequent profiles, 6 and 3, represent 11 and 6 million inhabitants respectively. This was expected to some extend by looking at the characteristics of their corresponding archetypoids. Profile 3 is formed by individuals with poor health but who nevertheless did not practice many health measures or stayed at home a lot, so it is not surprising that it is an unusual profile. On the other hand, the rareness of profile 6 is also not surprising, since in general it represents individuals who had a direct contact with COVID and in the first wave of the pandemic these people represented a small percentage of the European population.

In order to check the coherence between each group or cluster and the archetypoids that yield them, in Table 4 in the Appendix we provide some summary statistics for the clusters obtained, where it is confirmed that the individuals of each profile maintain the characteristics of the archetypoid that created them. In particular, observations belonging to profile 1 correspond to individuals with poor general, physical and mental health, little social interaction. It can be considered as “the worst” in terms of health deterioration and social isolation, what we call “COVID side effects”. Individuals in profile 2 have characteristics similar to Arch.2: good or fair health, but nevertheless a social life that has been greatly reduced by the outbreak. Profile 3 represents individuals with intermediate values of the indicators in almost all aspects, and like Arch.3, they have high values in health indicators (except for mental health), while the indicators of social relationships or practice of health measures are low compared to the other profiles. Profile 4 can be considered “the best” of all since it represents people in very good health and whom the pandemic did not affected too much in their social relationships or habits; Profile 5 represents individuals with low values in general in all indicators (both health and social life) but nevertheless they are individuals who changed their habits a lot and now practice many measures of safety against viruses. Finally, profile 6 is one of the most interesting ones, since it clearly represents individuals which experienced a direct contact with COVID, and in addition, it highlights its high values in mental health and healthcare. We give a summary of the main characteristics of the profiles in Table 3.

**Table 3:** Main characteristics of the profiles

Profile 1 (the worst)	Poor health, Poor mental health, High fear of leaving home,
	High change in habits and safety measures,
	High social isolation, Moderate electronic isolation.
Profile 2	Good or fair mental and physical health, High fear of leaving home
	High change in habits and safety measures,
	High social isolation.
Profile 3	Poor health, Little change in habits and safety measures,
	Low electronic isolation.
Profile 4 (the best)	Good health, Low fear of leaving home,
	Little change in habits and safety measures,
	High electronic isolation.
Profile 5	Good physical and mental health, Low fear of leaving home,
	Keep social contact, Low electronic isolation.
Profile 6	Poor mental health, High fear of leaving home,
	Low electronic isolation, Experienced COVID.

### Stringency index

**Fig. 3:**
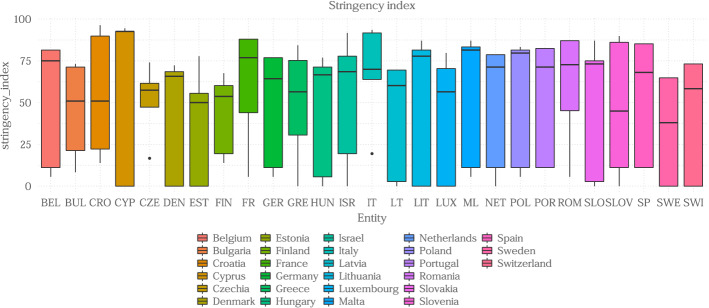
Stringency index in terms of country

We are interested in drawing conclusions from the results obtained by checking how both the archetypoids and their corresponding profiles are distributed across the different countries. First, it is necessary to know the context in which the individuals of each country lived, since in each region the pandemic affected in a different way and, above all, each State Member applied different restrictions. Therefore, we use the stringency index (Hale et al. 2021) to know the level of restrictions that directly affects the affected indicators. This index is a composite measure based on nine response indicators including school closures, workplace closures, and travel bans, rescaled to a value from 0 to 100 (100=strictest). In this case, we take into account only the data from the 27 European countries in our dataset and until June 1, which is the month in which the survey began. The distribution of the stringency index by country is plotted in Fig. 3.

From January to June 2020, in general the 27 countries experience a great variance of this index, since while at the beginning of 2020 no measures were taken against the virus with the March outbreak, most of the stringency indices rose a lot. However, there are differences between countries, where Italy stands out mainly since the virus arrived there much earlier and since January 2020 they maintained very high stringency measures. Although Fig. 3 shows the different values that this index took during these months, the most relevant value is the maximum of each of the countries, since in order to know the situation that its citizens lived, the important thing is to know how strong became the restrictions.

### Distribution of the profiles

First, we check by means of a bar plot how the different profiles calculated in the population are distributed by age group, gender and country. It is important to know if some profiles are more associated to some socio-demographic characteristics than others. For example, if the individuals who were most socially isolated or with poor health are men or women or even older or younger.

**Fig. 4:**
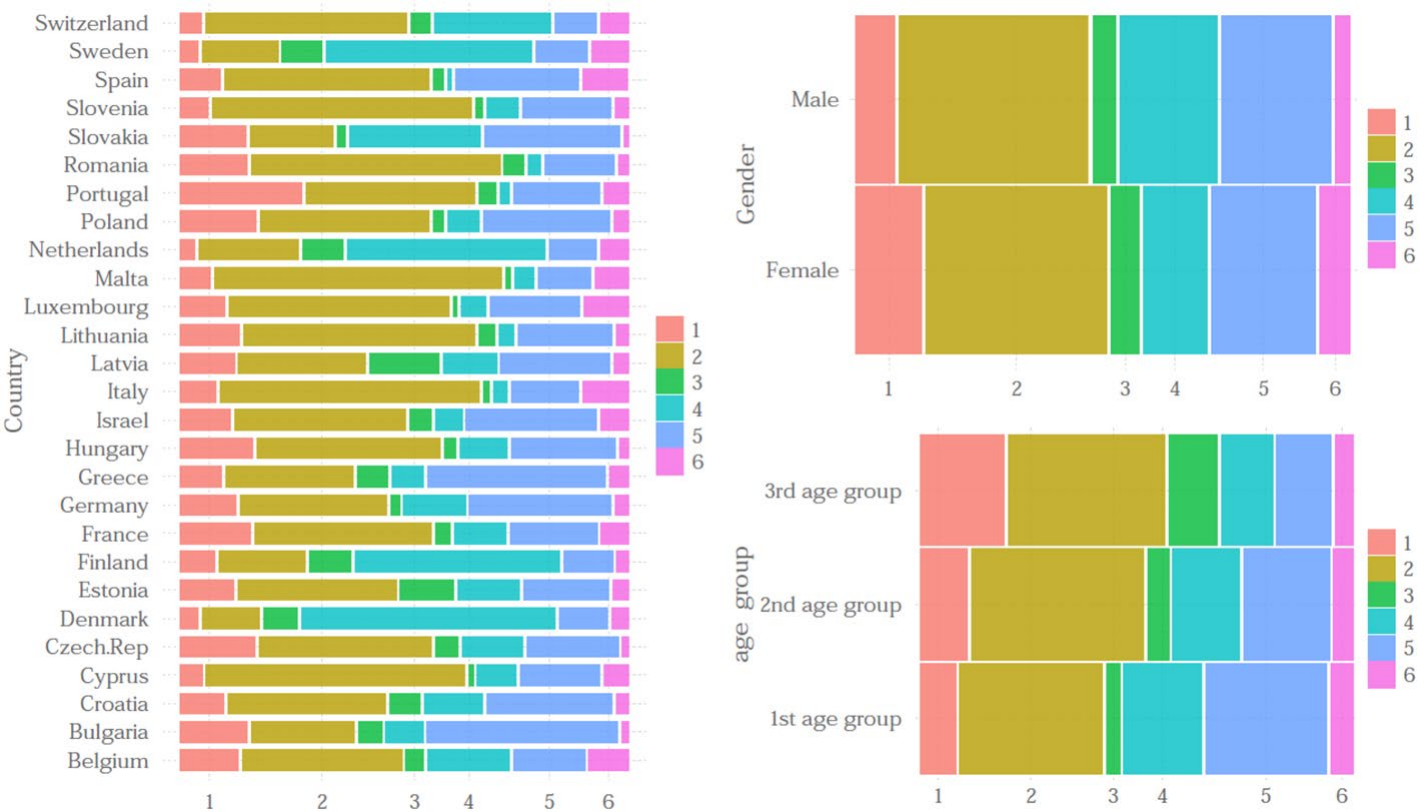
Distribution of profiles by country, gender and age group

As we saw in Fig. 2, profile 2 is the most frequent of all and therefore the most common in most countries. It stands out with very high frequencies in countries such as Italy or Malta, while it is a rare profile in Nordic countries like Denmark, Sweden or Finland (see Fig. 4). This profile is directly related to countries that have suffered great restrictions and therefore have high values in the stringency index (see Fig. 3). Profile 5, which the second most frequent, is very common in countries such as Bulgaria or Greece, while it is rare in the Netherlands and Switzerland. This profile is more present in those countries where security measures were taken against the virus despite not having major restrictions or not having many infections in the first wave of the pandemic. On the contrary, it is rare in countries where not many health safety measures were taken. Regarding profile 4, a great variance by country is observed. While in the Netherlands, Denmark or Sweden it represents almost half of its population, in Spain or Portugal it is only $1\%$ and $2\%$ respectively. Note that this profile is more frequent in countries that have a good health system and that did not suffer great restrictions in the first wave. Profile 1 is made up of individuals with poor health and whose social life has been reduced. Figure 4 shows us that it is a common profile in countries such as Portugal or the Czech Republic and rare in others like the Netherlands, Sweden, Switzerland or Denmark. It seems clear that this profile is residual in the richest and most developed countries of Europe. Profile 3, which is the least frequent of all, is more common in Estonia and Lithuania, followed by countries like the Netherlands or Sweden. It was to be expected that this profile would be common in these latter countries because, as we have already seen, their population has not suffered many restrictions and used too many security measures against COVID, but the high frequency value in the Baltic countries is striking. This finding may indicate that these countries have a higher percentage of people in poor health who have not been careful to catch the virus. Finally, profile 6, which is generally rare, gives us plenty of information about how each country suffered the first wave. Spain, Italy, Luxembourg and even Belgium stand out above all as countries in which it is more common, that is, it coincides with countries that experienced a serious health crisis and therefore there were more infections and healthcare for citizens worsened a lot.

Looking at the right top panel of Fig. 4, there do not seem to be great differences in the profiles by gender, except in the case of profiles 1 and 6, which are much more frequent in women. This finding may indicate, first, that within people with poor health and who suffered a strict lock-down, there are more women and, second, they also suffered more directly from COVID and the lack of healthcare. Conversely, profile 4 (which had the best characteristics) is more frequent in men than in women. In terms of age group, some differences are noteworthy. For instance, profiles 1 and 3 (which were made up of individuals with poorer health) tend to increase with age, whereas the contrary happen for profiles 4 and 5. As we said before, profile 2 is the most common in all age groups. However, it is important to pay special attention to the second most frequent profile in the oldest group (people aged 80 or over), which is profile 1, whose individuals experimented high social isolation apart from poor health, and fear of leaving home.

**Fig. 5:**
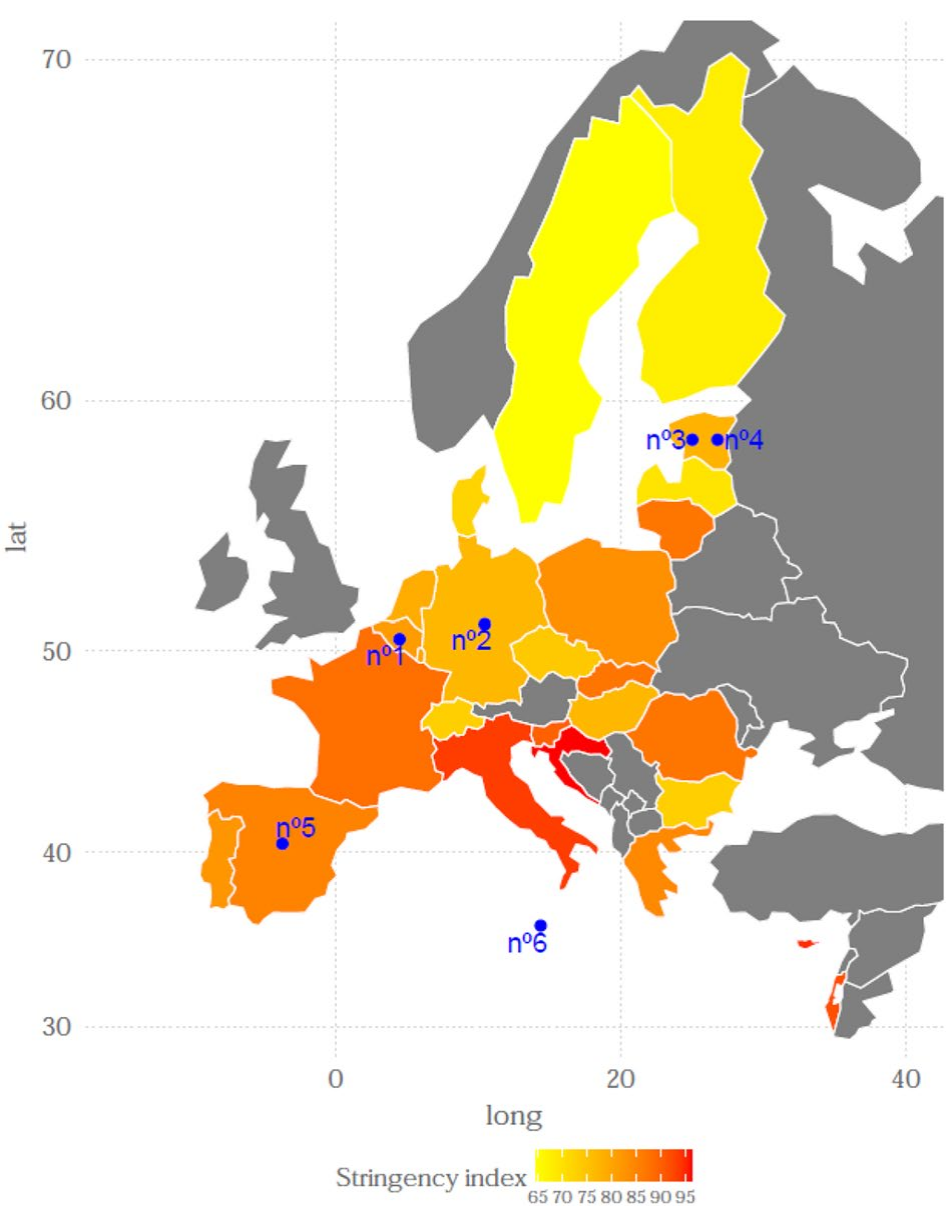
Geographical distribution of the archetypoids

Finally, in the left panel of Fig. 5 we depict the geographical location of the archetypoids, where countries are coloured according to its maximum level of the stringency during the months of January and June 2020. As it can be seen, Italy, Israel, Cyprus and Croatia stand out for their high value of stringency index, while the Nordic countries maintain very low values. The archetypoids are located in Belgium, Germany, Estonia, Spain and Malta.

### Geographical distribution of each profile

Before ending, we carry out an individual analysis of each profile in order to better understand their properties and how they are distributed across Europe. This is done by plotting the frequency of each profile per country in geographical maps. In particular, we carry out this study in profile pairs, which share some characteristic, in order to look for similarities in the geographical distribution of both profiles.

**Fig. 6:**
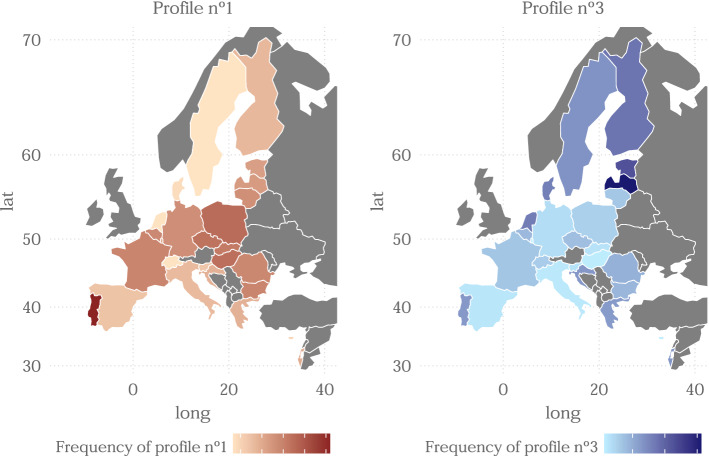
Geographical distribution of profiles 1 and 3

We start the study with profiles 1 and 3, these two groups have in common poor general and physical health. However, they differ in mental health (with very high values in the case of profile 1), and variables of security measures and social contact (which take also high values for profile 1). Figure 6 shows that there do not seem to be many similarities in the geographical distribution for this pair of profiles, except in the case of Portugal where in both cases each profile has a relatively high frequency. This may indicate in some way a poor state of health of the citizens of Portugal. On the other hand, profile 1 is seen perhaps more frequent in eastern European countries and profile 3 in Baltic countries, as we have seen in Fig. 4.

**Fig. 7:**
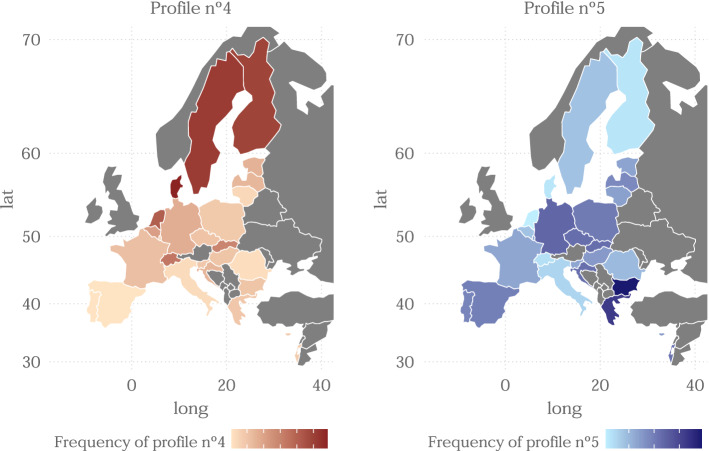
Geographical distribution of profiles 4 and 5

Analogously, in Fig. 7 we analyse profiles 4 and 5. Both profiles are made up of individuals in good health and that in general they have not greatly reduced their social contacts or undergone a strict lock-down, although profile 4 takes slightly higher values in these indicators, its only major difference is in the indicator of health safety measures, where profile 5 stands out clearly. As we said before, profile 4 is very common in the most developed and wealthy countries of Europe and in particular in the Nordic countries, it seems clear that in these countries COVID did not affect so seriously, at least by the time this survey was undertaken. However, profile 5 is more frequent in countries of southeastern Europe although it is more distributed throughout the map. Note that there does not appear to be any similarity in the distribution of these two profiles, indeed, it appears to have completely opposite distributions despite being similar groups and only differ greatly in one indicator.

**Fig. 8:**
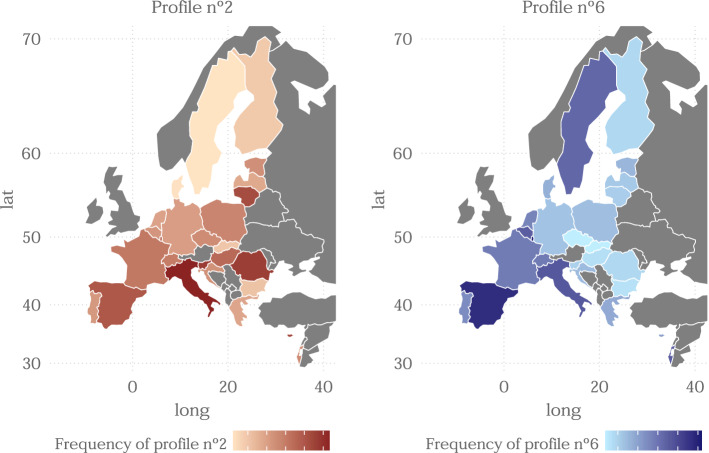
Geographical distribution of profiles 2 and 6

**Fig. 9:**
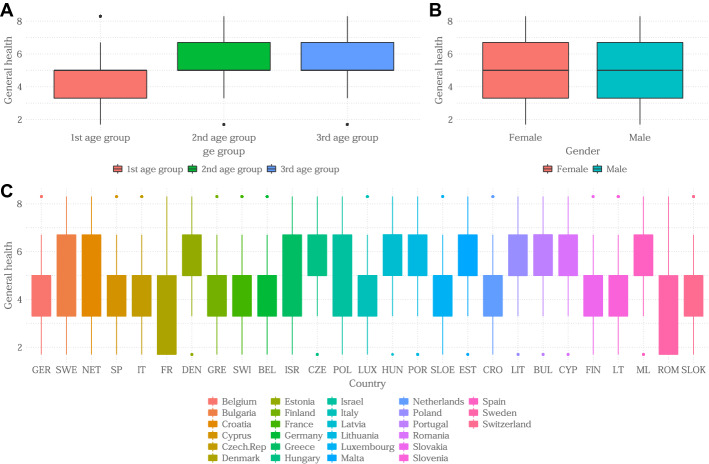
General health in terms of age group, gender and country

**Fig. 10:**
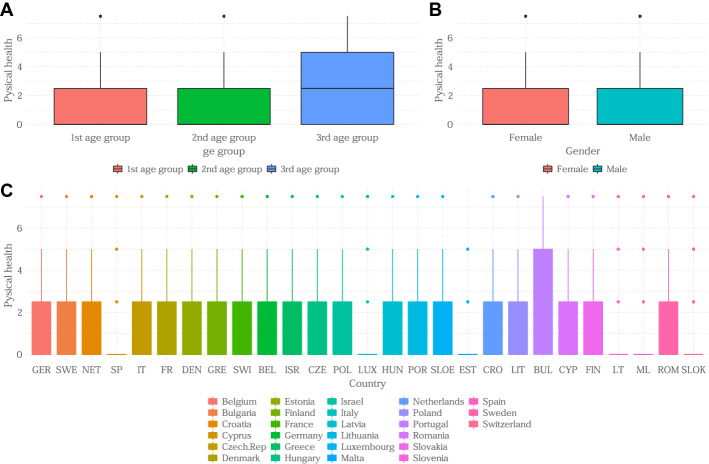
Physical health in terms of age group, gender and country

**Fig. 11:**
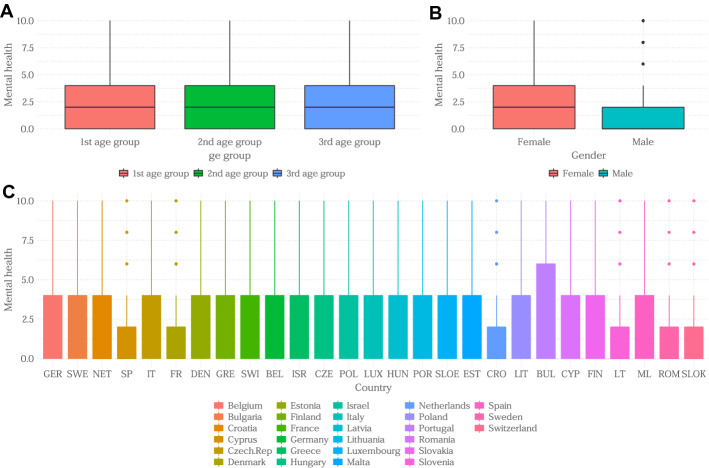
Mental health in terms of age group, gender and country

**Fig. 12:**
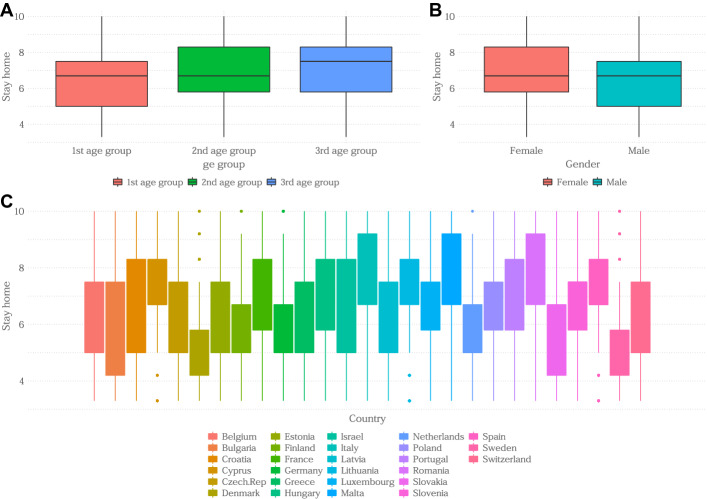
Stay at home in terms of age group, gender and country

**Fig. 13:**
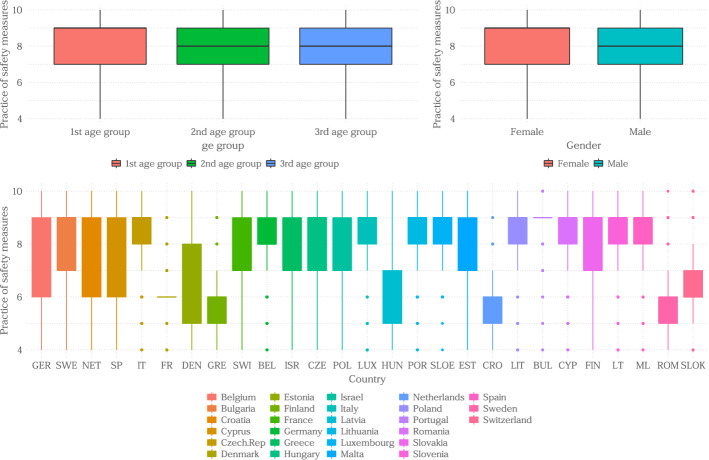
Practice of safety measures in terms of age group, gender and country

**Fig. 14:**
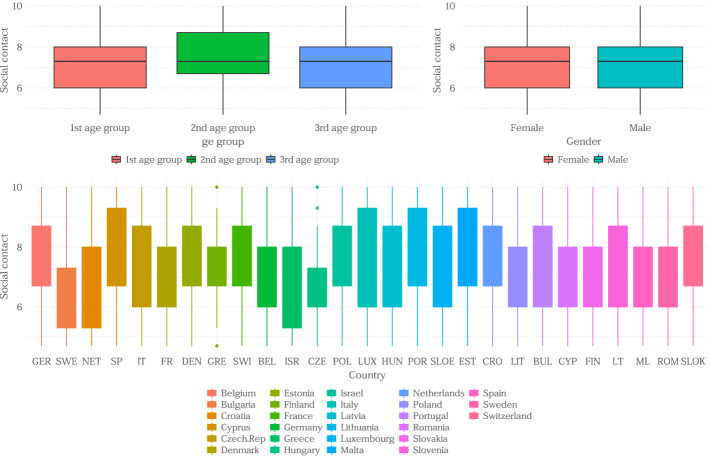
Social contact in terms of age group, gender and country

**Fig. 15:**
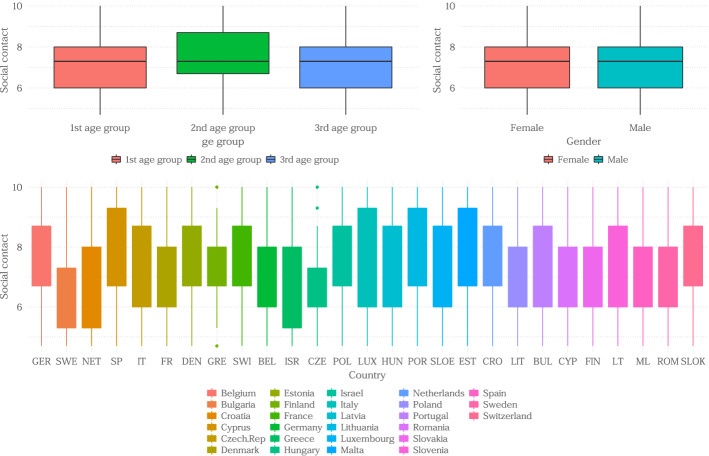
Electronic social contact in terms of age group, gender and country

**Fig. 16:**
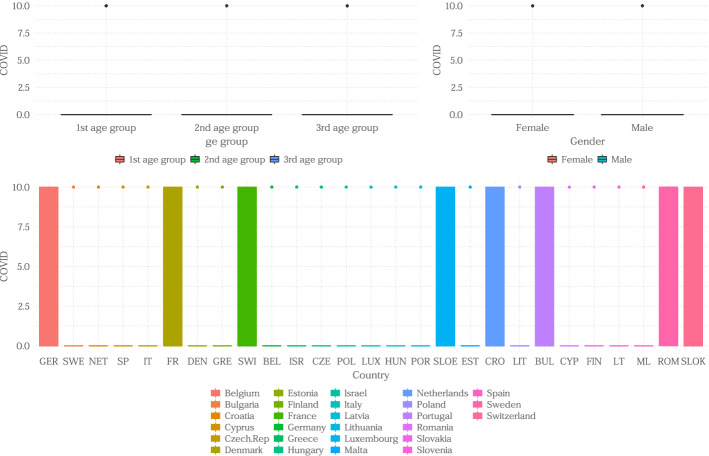
COVID in terms of age group, gender and country

**Fig. 17:**
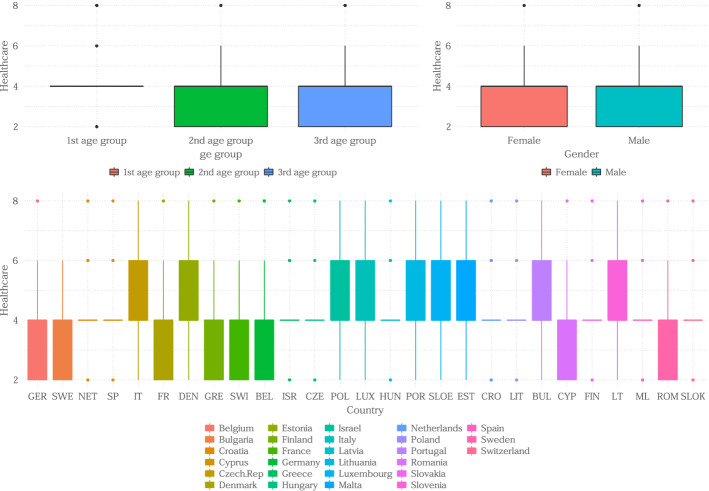
Healthcare in terms of age group, gender and country

**Fig. 18:**
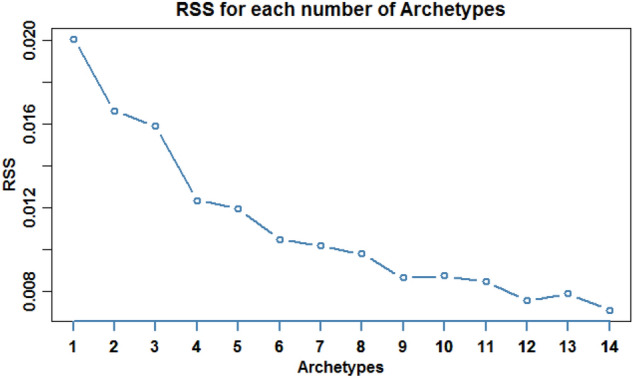
Scree-plot of RSS versus *k*

Finally, Fig. 8 shows the geographical distribution of profiles 2 and 6. Both profiles are formed by individuals with similar health (except mental health) and their individuals experienced enough social restrictions and measures security against COVID. However, they differ in mental health and especially in the indicators of COVID and healthcare since profile 6 stands out for it. Curiously, their geographical distributions do not differ much, since they may coincide in countries that had a high index of stringency, such as Spain and Italy. Profile 2 seems to have a similar geographical distribution to that of the stringency index (Fig. 5), probably since this profile is made up of individuals who underwent the most stringent lock-down. While profile number 6 is clearly distributed on countries with high number of COVID cases in the first wave or that worse manage the emergency. The comparison between the two maps of Sweden is striking, since it is one of the countries that imposed the least restrictions but nevertheless the virus affected the population and their healthcare a lot. Similar conclusions were reached by Primc and Slabe-Erker (2020).

## Conclusions

In this paper we propose an innovative approach, based on extremal observations, to obtain profiles or groups of homogeneus characteristics from survey data. One of the advantage is that the resulting profiles are easier to interpret than those estimated from central observations (like those obtained in other previous studies with SHARE database Grané et al. 2020, 2021). The protocol is based on three stages: First, composite indicators are designed, each of which focuses on a particular topic of interest; Second, the unsupervised statistical learning technique called archetypoidal analysis is applied on these indicators to obtain a few set of representative individuals (called archetypoids), and next the dataset is partitioned around the archetypoids by using Mahalanobis distance; Finally, the resulting clusters are analysed and visualized by means of other descriptive variables of interest, not included in the design of the indicators.

The methodology was illustrated on data coming from the first SHARE COVID-19 Survey, which was undertaken on people aged 50 and over living in 26 EU State Members plus Israel. Nevertheless, the method is wide enough to be applied to other surveys with large datasets and multivariate heterogeneous data.

As a result six clusters were found, and can be interpreted as health and behavioural profiles in coping with the COVID emergency. The two most frequent groups represent individuals with opposite characteristics, while in profile 2 individuals experienced great restrictions and their social life has been greatly reduced in profile 5 this has not occurred despite having practised preventive measures against the virus. These two profiles show us how the pandemic affected each individual depending on their country. Of special concern is profile 1, which is the second most frequent in the oldest group (people aged 80 or over), and whose individuals experimented high social isolation apart from poor health, and fear of leaving home.

In general terms, we can say that women stayed more at home and therefore suffered greater social distancing than men. In addition, women saw their healthcare deteriorate to a greater extent since the outbreak, although perhaps this is because they generally make more use of these services. On the other hand, as might be expected, older people are more present in the profiles with little social interaction and poor health, although in general they took more precautions against the virus by staying at home or taking health measures. Also unsurprisingly, younger people (although all aged 50 or over) were more carefree with the virus, leaving more of the home.

Although we have drawn important conclusions about the population based on their age or gender, the greatest differences are found according to the country or the geographical situation. For this, it is necessary to take into account the stringency index or the restrictions of each country. In general, in most of the surveyed countries, the inhabitants underwent great changes in their social lives, restricting themselves a lot and often living lock-downs. In this group, countries such as Italy, Spain or Belgium stand out, which were affected the most by the first wave of COVID. However, there is a clear trend and that is that in the northern European countries these restrictions were not as strict and the population did not suffer so much the consequences, in addition to the fact that its inhabitants did not practice too many precautionary measures. In this group are Sweden, Netherlands or Denmark, countries in which, in principle, the first wave was not so hard. A third group of countries can also be distinguished, in which the first wave did not affect as much, but both the institutions and the citizens themselves did take precautionary measures (although not as much as in the most affected countries).

It is necessary to comment separately on the conclusions drawn with profile 6, which is the one corresponding to people who experienced COVID closely. Despite the fact that in general this profile is more frequent in countries that experienced great social restrictions, such as Spain or Italy, and whose healthcare has also weakened (another of the characteristics of this profile), it is surprising how frequent this profile is in Sweden. Although it is a rich country, where, as we said, great prevention measures were not applied and its inhabitants did not practice too many security measures, we could think that the virus did not affect the population too much and that is why there was no careful. However, the frequency of this profile is high, which indicates that COVID did affect its population a lot and that its health system was affected.

Finally, this study allowed us to better understand how the first wave of COVID affected the countries of the European Union and Israel, in the population aged 50 or over. It also provides us with information about which countries better controlled the situation or which measures are efficient and they truly help slow the spread of the virus. For months, we have been focusing on data on infections and we have worried less about the consequences that this pandemic has on the life of the population, that is, its side effects. It is clear that, the measures imposed (“staying at home”) helped to reduce the rate of infection but also led to a worsening of pre-existing mental health problems, resulting in a negative impact on population’s well-being (anxiety, stress and other negative feelings and concerns about the practical implications of the response to the pandemic, including financial hardship). Thus, a balance between the potential benefits of enforced isolation and the psychological health costs seems necessary.

## Appendix

Here provide some plots for a more in-depth analysis of each indicator. For each indicator we provide conditional plots regarding age group, gender and country. Age was discretized into three groups: people between 50 and 65 years old, people between 65 and 80 years old, and people over 80 years old., Table

**Table 4:** Summary statistics for the profiles

	Gener. health	Phys. health	Mental health	Stay home	Safety measures	Social contact	E-Soc. contact	COV	Health-care
	*Profile 1: 22 million inhabitants aged 50 or over*								
$Q_1$	5.0	2.5	4.0	6.7	7.0	6.7	5.3	0.0	2.0
Median	6.7	5.0	6.0	7.5	9.0	8.0	6.0	0.0	4.0
Mean	6.5	4.8	6.3	7.4	8.1	7.7	6.1	1.1	4.2
$Q_3$	8.3	7.5	8.0	8.3	9.0	8.7	7.3	0.0	6.0
	*Profile 2: 73.2 million inhabitants aged 50 or over*								
$Q_1$	5	0.0	0.0	6.7	7	7.3	4.0	0.0	4.0
Median	5	0.0	0.0	7.5	9	8.0	5.3	0.0	4.0
Mean	5	0.7	1.3	7.7	8	8.2	5.5	1.3	4.1
$Q_3$	5	0.0	2.0	8.3	9	9.3	6.7	0.0	4.0
	*Profile 3: 6 million inhabitants aged 50 or over*								
$Q_1$	5.0	2.5	0.0	5.8	5.0	6.0	4.0	0.0	4.0
Median	6.7	5.0	2.0	6.7	6.0	7.3	5.3	0.0	4.0
Mean	6.6	5.1	1.5	7.1	5.9	7.0	5.1	1.5	4.2
$Q_3$	6.7	7.5	2.0	8.3	7.0	8.0	6.0	0.0	6.0
	*Profile 4: 26 million inhabitants aged 50 or over*								
$Q_1$	1.7	0.0	0.0	4.2	5.0	6.0	6.0	0.0	4.0
Median	3.3	0.0	0.0	5.0	6.0	7.3	6.7	0.0	4.0
Mean	3.7	0.8	1.3	5.3	5.9	7.1	6.7	1.6	4.1
$Q_3$	5.0	0.0	2.0	5.8	7.0	8.0	7.3	0.0	4.0
	*Profile 5: 49 million inhabitants aged 50 or over*								
$Q_1$	5.0	0.0	0.0	4.2	7	5.3	3.3	0.0	4.0
Median	5.0	0.0	0.0	5.0	8	6.0	4.7	0.0	4.0
Mean	5.0	0.9	1.4	5.3	8	6.2	4.5	1.3	4.1
$Q_3$	6.7	2.5	2.0	5.8	9	7.3	5.3	0.0	4.0
	*Profile 6: 11 million inhabitants aged 50 or over*								
$Q_1$	3.3	0.0	4.0	6.7	6.0	6.0	4.0	0.0	4.0
Median	5.0	0.0	6.0	7.5	8.0	7.3	4.7	10.0	4.0
Mean	5.1	0.9	6.1	7.2	7.6	7.2	4.7	6.8	5.0
$Q_3$	6.7	2.5	8.0	8.3	9.0	8.0	5.3	10.0	6.0

^4^

## Footnotes

1 The outbreak of COVID-19 hit SHARE in the middle of its 8th Wave of data collection and it had to be suspended in all participating countries in March 2020. After the end of lock-down, in mid-June 2020 the survey of individuals from the 27 countries included in the database was retaken (Börsch-Supan 2020).
